# Calculs vésicaux multiples compliquant une fistule vésico-vaginale d’origine tuberculeuse: à propos d’un cas exceptionnel

**DOI:** 10.11604/pamj.2019.33.126.16674

**Published:** 2019-06-19

**Authors:** Mustapha Ahsaini, Youssef Kharbach, Hamid Azelmad, Soufiane Mellas, Jalaleddine Elammari, Mohammed Fadl Tazi, Mohammed Jamal Elfassi, Moulay Hassan Farih, Mohammed Sekal, Taoufik Harmouch

**Affiliations:** 1 Service d'Urologie, Centre Hospitalier Universitaire Hassan II de Fès, Fès, Maroc; 2 Service d'Anatomopathologie, Centre Hospitalier Universitaire Hassan II de Fès, Fès, Maroc

**Keywords:** Calcul vésical, fistule vesico-vaginale, tuberculose urogénitale, antituberculeux, chirurgie, Bladder stone, vesicovaginal fistula, urogenital tuberculosis, antituberculous drugs, surgery

## Abstract

La fistule vésico-vaginale (FVV) continue d'être un problème majeur de la santé publique dans les pays en développement. Devant la situation particulière d'association FVV avec calcul; la question qui se pose d'abord est de définir si la fistule est primaire ou secondaire au calcul vésical, ensuite s'il faut traiter en un seul temps ou en deux temps. Mais la particularité de notre observation c'est l'origine tuberculeuse de cette présentation clinique rarissime. Il s'agit d'une patiente âgée de 62 ans qui a comme antécédent une spondylodisciite tuberculeuse traitée et déclarée guéri il y'a 4 ans, qui présentait depuis 2 ans des fuites urinaires permanente. L'examen clinique trouve une synéchie vaginale presque complète. Le complément scannographique et cystographique a mis en évidence la présence de 3 calculs sur le trajet de la FVV dont le plus grand mesure 6cm de grand axe avec passage du produit de contraste à la cavité utérovaginale à travers la fistule. La patiente fut admise au bloc opératoire où elle a bénéficié d'une cystolithotomie avec ablation des calculs qui se trouvaient sur le trajet fistuleux avec la biopsie du trajet fistuleux et la fermeture de la FVV en deux plans sans interposition de tissu autologue en un seul temps, les résultats anatomopathologiques ont confirmé la présence d'une tuberculose active sur le trajet fistuleux ayant nécessité une reprise de traitement anti-bacillaire pendant 9 mois. Le contrôle à 3 mois, 6 et à 9 mois a objectivé une bonne évolution clinique avec absence de fuites urinaire. L'origine tuberculeuse d'une FVV associée à des calculs urinaires est très peu retrouvée dans la littérature. Notre cas démontre la faisabilité d'un traitement médical anti-bacillaire associé à un traitement chirurgical en un seul temps avec des résultats très satisfaisants malgré les antécédents de notre patiente et l'ancienneté de sa pathologie.

## Abstract (en)

Vesicovaginal fistula (VVF) continues to be a major public health problem in developing countries. Given the particular association of VVF with stones, the question that arises is whether the fistula is primary or secondary to bladder stone and then whether to use single-stage or two-stage treatment. But what is special about this study is that these rare clinical features are due to tuberculosis. We here report the case of a 62-year old female patient with a history of treated tuberculous spondylodiscitis. The patient had been declared cured 4 years before. For the previous 2 years she had been suffering from continuous urinary incontinence. Clinical examination showed almost complete vaginal synechia. Complementary scannography and cystography showed the presence of 3 stones on the way to the VVF. The largest stone measured 6cm along its longer axis with passage of contrast material into the uterovaginal cavity through the fistula. The patient was admitted to the operating room where she underwent cystolithotomy with ablation of the stones that were on the way to the fistula, biopsy of the fistulous tract and single-stage closure of the VVF in two layers without interposition of the autologous tissue. Anatomopathological results confirmed the presence of active tuberculosis on the way to the fistula, requiring resumption of antibacillary treatment for 9 months. During the follow-up visit at 3, 6 and 9 months the patient showed good clinical status with absence of urinary incontinence. VVF secondary to tuberculosis associated with urinary stones appears very little in literature. Our case demonstrates the feasibility of antibacillar medical treatment associated with single-stage surgical treatment with very satisfactory results despite the history of our patient and the duration of his disease.

## Introduction

La fistule vésico-vaginale (FVV) continue d'être un problème majeur de la santé publique dans les pays en dévéloppement, dont la majorité est due à un accouchement dystocique [1] et l'origine tuberculeuse est peu retrouvée dans la littérature [2]. Rarement, les patientes avec la FVV peuvent développer des calculs urinaires [3]. Ces calculs vésicaux peuvent se former en raison d'une infection urinaire, d'une déshydratation, de corps étrangers, d'une stase urinaire ou de contaminants provenant de sources extérieures [4]. Devant la situation particulière d'association FVV avec calcul; la question qui se pose est de définir si la fistule est primaire ou secondaire au calcul vésical, puis s'il faut traiter en un seul temps ou en deux temps. La discussion de notre cas illustre parfaitement le sujet. Au meilleur de nos connaissances c'est le premier cas dans la littérature qui associe des calculs vésicaux avec une fistule vésico-vaginale d'origine tuberculeuse.

## Patient et observation

**Figure 1:**
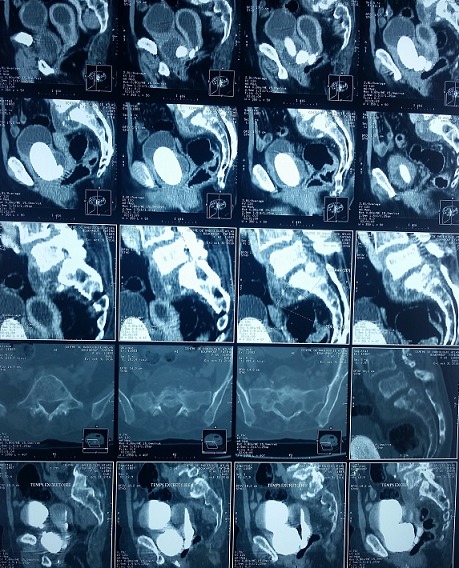
Image scannographique montrant atteinte lytique et condensante du sacrum et du promontoire avec une collection présacrée; à noter la présence de calcifications (flèches rouges) au sein de cette collection hautement suggestive de leur origine tuberculeuse; scannographique avec des reconstructions longitudinales sans injection de produit de contraste montrant 3 calculs au niveau de la FVV; scannographique avec des reconstructions longitudinales avec injection de produit de contraste montrant la FVV

**Figure 2:**
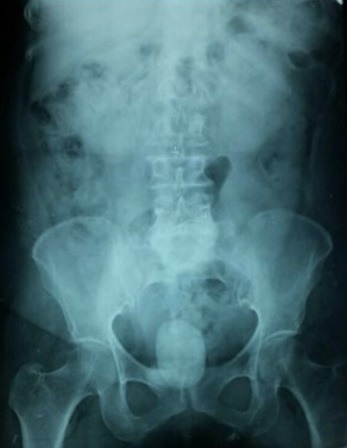
Image d’AUSP montrant un gros calcul se projetant au niveau de l’aire vésical

Il s'agit d'une patiente âgée de 62 ans qui a comme antécédent une spondylodisciite tuberculeuse (Figure 1), traitée et déclarée guérie il y'a 4 ans. La patiente présentait depuis 2 ans des fuites urinaires permanentes par le vagin avec des cystalgies intermittentes sans autres signes associés. L'examen clinique a trouvé une patiente en bon état général. La patiente avait une synéchie vaginale qui a rendu impossible l'examen gynécologique, mais nous avons néanmoins pu visualiser des fuites urinaires permanentes par l'orifice vaginal au dessus du col vésical (> 3cm du méat urétral). Nous avons réalisé un arbre urinaire sans préparation (AUSP) (Figure 2) qui a montré la présence d'un gros calcul se projetant au niveau de l'aire vésical. La cystographie a montré une FVV avec passage important du produit de contraste de la vessie à la cavité utérovaginale. Le complément scannographique (cysto-scanner) a mis en évidence la présence de 3 calculs sur le trajet de la FVV dont le plus grand mesure 6cm de grand axe avec passage du produit de contraste à la cavité utérine à travers la fistule vésico-vaginale (Figure 1). La fonction rénale était correcte et l'examen cytobactériologique des urines était négatif.

**Figure 3:**
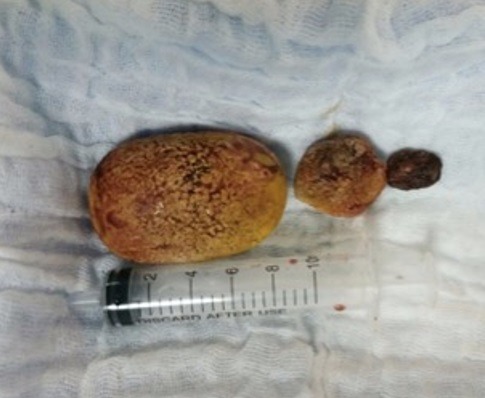
Image des calculs après extraction

**Figure 4:**
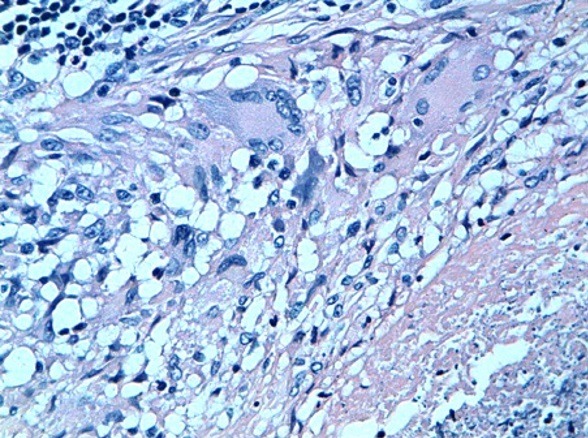
Aspect histologique de la biopsie de la fistule vésico-vaginale montrant des lésions granulomateuses épithélioides avec des cellules géantes de type Langhans et une nécrose caséeuse (coloration hémateine-éosine, Grossissement 200)

**Figure 5:**
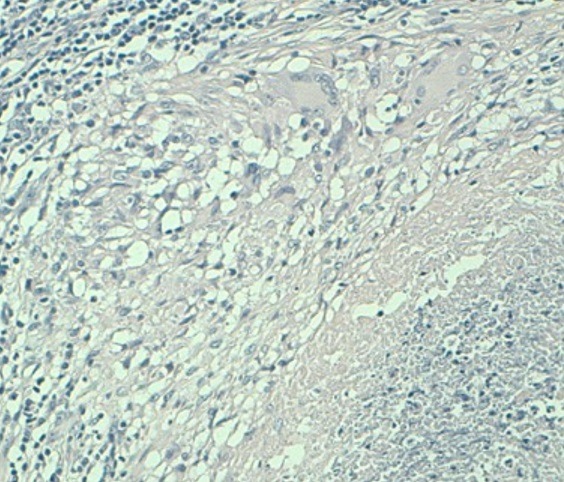
Même aspect histologique avec un fort grossissement (400)

Devant cette situation nous nous sommes posé deux questions: *s'agit-t-il d'un calcul compliquant une FVV ou la FVV était-elle secondaire au calcul?* D'autant plus qu'il s'agit d'un antécédent de tuberculose uro-génitale. *Allons-nous faire l'ablation du calcul en premier puis procéder à la cure de la FVV dans un 2^ème^ temps ou traiter le tout en un seul temps?* Après une revue de la littérature, nous avons opté vers un traitement en un seul temps. La patiente fut admise au bloc opératoire où nous avons réalisé une cystolithotomie dont l'exploration a trouvé que la FVV siégeait un peu au-dessus de la barre inter urétérale avec extraction laborieuse des calculs qui se trouvaient sur le trajet fistuleux (Figure 3) et la réalisation d'une biopsie du trajet fistuleux avec fermeture de la FVV en deux plans (plan vaginal et plan vésical) sans interposition de tissu autologue. Ceci a été réalisé par chirurgie ouverte par laparotomie médiane sous ombilicale. Les suites postopératoires étaient simples avec ablation de la sonde vésicale à j15. L'analyse anatomopathologique de la biopsie du trajet fistuleux a objectivé lésions granulomateuses épithélioides avec des cellules géantes de type Langhans et une nécrose caséeuse correspondant à une cystite tuberculeuse (Figure 4 et Figure 5). La patiente a bénéficié d'un traitement anti-bacillaire sur une durée de 9 mois, elle a été revue à 3, 6 et 9 mois, elle n'a plus de fuites urinaires.

## Discussion

La fistule vésico-vaginale d'origine obstétricale est un motif important d'hospitalisation dans les pays en voie de développement en particulier en Afrique [5]. La plupart de ces femmes appartiennent à des classes socio-économiques défavorisées et le problème est aggravé par la sous-médicalisation dans ces pays. Dans de nombreux cas, le problème est aggravé par l'abandon des patientes par leur famille. Ces facteurs entraînent un retard de la prise en charge de ces patientes [5, 6]. Dalela *et al.* à trouvé un intervalle d'un moyenne 28,8 mois après l'apparition de la fistule avant que les patientes ne partent consulter, principalement lorsque la maladie est devenue douloureuse et difficile à gérer [5]. Sur le plan mondial, au moins 3 millions de femmes ont une fistule non traitée, alors qu'entre 30 000 et 130 000 nouvelles fistules se développent chaque année en Afrique [6]. La fermeture spontanée des fistules a été décrite mais la vitesse avec laquelle cela se produit est susceptible d'être sous-estimée en raison du fait que ces cas sont rarement référés pour un traitement ultérieur [7]. L'association entre FVV et calcul de vessie suscite des questions diagnostique et thérapeutique. Car, les calculs de la vessie sont habituellement secondaires à la stase urinaire dans les obstructions sous vésicales ou à une origine infectieuse, quand ils augmentent de volume, ils peuvent éroder la paroi vésicale en provoquant une nécrose ischémique, entraînant une fistule vésicale spontanée [8]. Mais des fois, et malgré la rareté de la FVV, on peut avoir des patientes « fistuleuses » qui vont développer des calculs vésicaux ou vaginaux [3, 5].

Des descriptions de fistules vésico-vaginales tuberculeuses comme le cas de notre patiente, ont été difficiles à trouver dans la littérature [2, 9]. Notre cas est tout aussi atypique, car, à notre avis, l'antécédent de spondylodisciite tuberculeux a été un élément clé faisant suspecter le diagnostic d'une cystite tuberculeuse avec la réalisation en peropératoire d'une biopsie du trajet fistuleux. Le traitement de ces patientes suscite lui aussi une réflexion entre deux options thérapeutiques: une procédure en deux temps, souvent préconisée pour la réparation de la fistule avec des calculs vésicaux associés. L'ablation des calculs est effectuée en premier, suivie après au moins 3 mois de la cure de la fistule dans un deuxième temps. Cette procédure trouve sa justification dans le fait que le plus souvent il y a une infection urinaire associée en plus de l'inflammation de la muqueuse vésicale due au calcul, le tout entrainera un retard voire un défaut de la cicatrisation [1, 4, 10]. Par contre, Shephard [1] a rapporté dans sa série 87 patientes présentant des calculs vésico-vaginaux compliquant une FVV, 51 cures de la fistule avec ablation du calcul en un seul temps. Ses résultats montrent que la réparation simultanée est possible et bénéfique.

Pour les femmes chez qui la cure est réussie, ce protocole réduit considérablement la souffrance et accélère le retour à la vie normale, car une réparation simultanée réussie entraîne un retour plus rapide à la continence qu'une réparation par étapes [1]. Cette étude montre aussi que le siège de la FVV est un indicateur pronostique important, les fistules haute et moyenne vaginale sont beaucoup plus susceptibles d'être fermées avec succès que les grosses fistules ou bas situées [1]. Dalela [4] a trouvé dans sa cohorte où toutes les fistules ont été réparées 3 mois après l'extraction du calcul un taux de réussite de 84,2%. Cependant, ses patientes avaient de petites fistules (1,9cm). C'est après avoir vu les résultats de Shephard que nous avons opté vers une cure en un seul temps qui a été un succès malgré la difficulté technique chez notre patiente. Afin d'obtenir des résultats optimaux, il nous semble évident que ces interventions chirurgicales doivent être réalisées par une équipe expérimentée dans un centre ayant une infrastructure adéquate. Selon Hillary [7] une expérience chirurgicale limitée semble augmenter les risques d'échec chirurgical. Notre geste chirurgical a été associé à la réalisation d'une biopsie vésicale qui a objectivé le diagnostic d'une cystite tuberculeuse qui fait la particularité de nôtre observation et qui a nécessité bien sur par la suite la mise en place d'un traitement médical antituberculeux pendant une durée de 9 mois avec une bonne évolution clinique et biologique.

## Conclusion

La FVV et la tuberculose restent malheureusement assez fréquentes dans les pays en voie de dévéloppement particulièrement en Afrique. La discussion du cas de notre patiente soulève des questions d'ordre diagnostique et thérapeutique qui n'ont pas encore fait l'unanimité dans la littérature internationale.

## Conflits d’intérêts

Les auteurs ne déclarent aucun conflit d'intérêts.
